# ‘If You Don't Speak Up, It Will Eat You Up’: A Critical Discourse Analysis of Voice and Silence in a Multicultural Clinical Healthcare System

**DOI:** 10.1111/nin.70159

**Published:** 2026-08-25

**Authors:** Shaista Salman Guraya, Farah Ennab, Hafidh Mohammad Khamis AlRajaby, Ibrahim Inuwa, Entesar Al Hammadi, Manal Al Halabi, Nabil Zary

**Affiliations:** ^1^ Institute of Learning Mohammed Bin Rashid University of Medicine and Health Sciences, Dubai Health Dubai UAE; ^2^ Emergency Department Rashid Hospital, Dubai Health Dubai UAE; ^3^ College of Medicine Mohammed Bin Rashid University of Medicine and Health Sciences, Dubai Health Dubai UAE; ^4^ Office of the Chief Clinical Officer, Patient Safety & Quality Department Dubai Health Dubai UAE; ^5^ Hamdan Bin Mohammed College of Dental Medicine Mohammed Bin Rashid University of Medicine and Health Sciences, Dubai Health Dubai UAE

**Keywords:** critical discourse analysis, health professions education, multicultural healthcare, qualitative research, speaking up

## Abstract

Despite sustained efforts to promote speaking up in healthcare, improvements remain ephemeral. In diverse clinical learning and practice environments shaped by generational, cultural, professional and pedagogical plurality, speaking up operates as a situated social practice. Adopting an interpretive bricolage, we conducted a critical discourse analysis of interviews with 17 healthcare professionals within a multicultural academic health system. We identified four definitional discourses and eight interpretive repertoires through which participants legitimised voice using different moral and relational logics, including loyalty, conscience, professionalism, discernment, relational navigation, procedural alignment and emotional authenticity. Only one repertoire, most prominent among residents, constructed speaking up as self‐combustion, where voicing concerns threatened professional survival. These findings suggest that dominant solutions centred on communicative competence and psychological safety are incomplete. Stewardship‐based repertoires enabled voice without reliance on either construct, while ‘wasta’ functioned not as corruption but as relational social capital enabling culturally intelligible action. Speaking up, therefore, emerged as a chameleon concept whose meaning shifts across contexts. Attempting to capture speaking up through another abstract construct risks a shotgun approach. Rather than treating voice as a discrete behaviour to be taught, speaking up may be better understood as a situated judgement shaped by hierarchy, legitimacy and relational consequence.

## 1 Introduction

Speaking up is commonly framed in healthcare as a behaviour that healthcare professionals (HCPs) either perform or fail to perform. Yet the continued difficulty of producing this behaviour, despite sustained intervention efforts, suggests a more basic problem. The meaning of speaking up itself may not be settled. Speaking up has become a central construct within contemporary patient safety discourse, widely positioned as a desirable professional behaviour that HCPs are expected to enact to prevent harm and improve care quality (Dyne et al. 2003; D. Schwappach and Richard 2018; van Dongen et al. 2024). Within this framing, failures of communication are interpreted as preventable safety lapses, and voice is treated as both a moral and professional obligation. Over time, this positioning has institutionalised speaking up as a behavioural expectation analogous to hand hygiene or checklist compliance, something HCPs should reliably perform when risk is perceived (Seo and Lee 2024). Unsurprisingly, the field is now replete with interventions designed to increase voice (Ginsburg and Bain 2017; Jones et al. 2021; Kepplinger et al. 2024; S. Kim et al. 2020; O'Donovan and McAuliffe 2020; Parker et al. 2019; Violato et al. 2024). Communication‐skills training, escalation pathways, reporting systems, leadership development and psychological safety initiatives are commonly treated as mechanisms capable of producing the desired behaviour (Okuyama et al. 2014). Multiple systematic reviews have synthesised this work, yet a consistent pattern remains. Gains are modest, uneven and difficult to sustain (Jones et al. 2021; Kepplinger et al. 2024; S. Kim et al. 2020; O'Donovan and McAuliffe 2020; Parker et al. 2019). Implementation science similarly cautions that education alone rarely changes behaviour without a receptive context and appropriate facilitation (Brown and McCormack 2016). This repeated intervention fatigue raises a more basic question: if speaking up is a teachable behaviour, why does it remain resistant to durable change?

Empirical studies already suggest that the absence of voice cannot be equated with the absence of concern (Beament and Mercer 2016; Creese et al. 2021; Landgren et al. 2016; Martinez et al. 2017). HCPs actively judge when silence is safer, more professional or more appropriate, weighing reputational risk, relational consequences and perceived futility. Notably, healthcare workers may speak readily for patient safety while remaining silent about their own working conditions, indicating that voice functions as a morally differentiated practice rather than a single behavioural tendency (Creese et al. 2021). Such findings challenge assumptions that increasing opportunities for voice necessarily reduces silence and instead point toward variation in how professionals interpret the act itself. What counts as legitimate voice, what silence signifies and what it costs to be heard appear to vary across hierarchy, profession and setting. Although contemporary evidence recognises individual, relational, contextual and organisational influences (van Dongen et al. 2024), expanding the list of predictors has not fully clarified the phenomenon itself. Increasingly comprehensive interventions have followed, yet without consistent success, which may reflect escalating intervention complexity without resolving underlying conceptual ambiguity (Okuyama et al. 2014). Organisational scholarship further complicates this behavioural framing by conceptualising voice as the outcome of competing motivational and inhibiting forces operating simultaneously at individual, relational and structural levels (van Dongen et al. 2024). Prosocial commitment, perceived efficacy, anticipated relational risk, power asymmetry, fear, futility and beliefs about hierarchy interact dynamically in shaping decisions to speak or remain silent (Morrison 2014; Morrison 2023). Psychological safety represents only one element within this broader calculus and does not by itself determine whether voice occurs (O'Donovan and McAuliffe 2020; O'Leary 2016). Individuals may remain silent despite perceived safety when speaking appears inappropriate or pointless, while others may speak despite risk when obligation or responsibility dominates. Despite this complexity, much of the healthcare literature continues to measure frequency, antecedents and outcomes of speaking up or evaluate strategies intended to increase it (Creese et al. 2021; Jones et al. 2021; Kepplinger et al. 2024; S. Kim et al. 2020; Morrison 2014; O'Donovan and McAuliffe 2020; Parker et al. 2019).

Comparatively, little work examines how HCPs themselves understand what they are doing when they ‘speak up’. Without such understanding, existing interventions risk attempting to modify behaviour whose meaning is neither uniform nor agreed upon. This ambiguity becomes particularly salient in contemporary healthcare environments characterised by increasing diversity, not only generational but cultural, professional and pedagogical (Flott and Linden 2016). Multicultural teams comprising physicians, nurses, pharmacists, therapists and trainees operate within differing norms of respect, responsibility, communication and authority. In such contexts, voice cannot be assumed to operate as a culturally neutral act, nor silence as a straightforward deficit. From a social constructionist perspective, meanings emerge through interaction rather than residing within individuals (Habib et al. 2025; Rees et al. 2020). Practices become intelligible through the discourses available within a community, shaping what can be said, by whom and with what consequences. However, HCPs do not merely reproduce these norms; they interpret and negotiate them in relation to responsibility, hierarchy and risk. Speaking up may therefore be better understood not solely as an internal disposition but as a socially negotiated practice constituted in interaction (Kincheloe and McLaren 2011).

Against this backdrop, we undertook a critical discourse analytic study to examine how HCPs in a multicultural academic healthcare setting construct the meaning, morality and legitimacy of speaking up. A discursive approach enables examination of how professionals negotiate voice within specific social contexts rather than measuring whether it occurs. Our aim was not to test behavioural hypotheses but to generate an interpretive account of how HCPs position speaking up in practice. Specifically, we aimed to explore how HCPs working in the United Arab Emirates (UAE) defined speaking up, justified or questioned it and how they constructed risk and responsibility.

## 2 Methodology

### 2.1 Study Design and Setting

This study was conducted within Dubai Health, a newly integrated academic health system in the UAE. Across its hospitals and ambulatory centres, clinical practice is enacted by culturally diverse and internationally trained professionals working within layered hierarchies of supervision, evaluation and authority. To anchor our study within this broader institutional context, we sampled two clinical sites that sit within this wider system: (1) a large public tertiary care hospital and (2) a specialised dental training institution. In this current study, we attempted to explore speaking up using a social constructionist (Liebrucks 2001; Rees et al. 2020) and a critical discourse‐analytic methodology (Kincheloe and McLaren 2011) rather than a behaviourist or positivist one. Speaking up, as a phenomenon embedded in culture, hierarchy and professional identity, cannot be reduced to individual attitudes or psychological traits. Instead, its meaning is shaped through language, interaction and culturally available interpretive resources. Our aim was therefore not to locate a singular, objective, universal essence of ‘speaking up’, neither to treat it as a stable internal disposition residing within individuals (Habib et al. 2025; Liebrucks 2001; Rees et al. 2020). We rejected reductionist assumptions that complex social practices can be explained through underlying traits, linear causal models or that there exists a natural, rational pattern to be uncovered through empirical testing.

To understand this, we proposed the following research question: How do HCPs in a multicultural academic healthcare setting discursively construct the meaning, morality and legitimacy of speaking up when describing their intentions to voice concerns or remain silent?

### 2.2 Epistemological Stance

We approached speaking up as multiple, contingent and situationally constructed, consistent with a social constructionist epistemology (Liebrucks 2001) informed by both interpretive and critical traditions (Kincheloe and McLaren 2011). Meanings were understood to arise from the discourses available within a multicultural healthcare system, rather than from essential psychological drives or fixed professional norms, while recognising that professionals are active interpreters who negotiate, resist and reshape these discourses as they make sense of professional responsibility, risk, identity and legitimacy within a social system. This orientation enabled attention to both participants' situated meaning‐making and the broader cultural, professional and institutional conditions through which such meanings became possible. Consistent with a bricoleur orientation, the object of study was approached through *webs of relationships* rather than as a *discrete phenomenon* in itself. Attention was therefore directed toward the processes, relationships and interconnections through which speaking up was constructed across personal, interpersonal, organisational and cultural levels (Kincheloe 2005). In line with Foucault's (1976) view (Power 2011), the social world through language, norms and cultural scripts shapes what can be said, by whom and with what consequences; however, these possibilities are taken up and made meaningful through situated reasoning.

### 2.3 Participants

Seventeen HCPs participated in this study, representing a wide range of ages (26–65 years), clinical disciplines, nationalities and cultural trajectories. The sample included junior trainees, mid‐career and senior HCPs, capturing the perspectives of those at varying stages of their identity formation. Participants included Emirati nationals, Arab HCPs raised or trained in Western contexts, long‐term expatriate practitioners from South Asia and professionals enculturated within the Gulf region. This diversity was analytically generative, allowing insight into the multiple moral and cultural resources through which speaking up was understood and enacted in everyday clinical practice (Table 1).

**Table 1:** Professional characteristics of participants in this study.

Participant	Gender	Clinical speciality	Nationality	Role seniority
A	F	EM	IND	J
B	M	Den	LIB/UK	S
C	F	IM	UAE	S
D	F	Nurs	UAE	M
E	F	Surg	UAE	J
F	M	Ortho	SYR/USA	J
G	F	Den	UAE	S
H	F	EM	AFG/SWE	J
I	F	EM	PAL	J
J	F	IM	IND	M
K	F	EM	UAE	S
L	F	EM	SYR	J
M	M	Den	UAE	M
N	M	Den	UAE	S
O	F	Nurs	IND	S
P	M	EM	IND	M
Q	F	Den	IRN	S

*Note:* Role seniority refers to stage of career, categorised by the number of years since commencing professional training—junior: < 5 years; mid‐career: 6–12 years, and senior: > 13 years.

Abbreviations: AFG, Afghanistan; Den, dental; EM, emergency medicine; IM, internal medicine; IND, India; IRN, Iran; J, junior; LIB, Libya; M, mid‐career; Nurs, nursing; Ortho, orthopaedic surgery; PAL, Palestine; S, senior; Surg, surgery; SWE, Sweden; SYR, Syria; UAE, United Arab Emirates; UK, United Kingdom; USA, United States of America.

### 2.4 Data Collection

This study formed part of a larger grant‐funded project examining cultural perceptions and speaking‐up behaviours using a validated survey instrument (reported elsewhere). Following completion of that phase, participants who indicated willingness to elaborate on their responses were invited to participate in the present qualitative study. A convenience‐based volunteer sampling approach was therefore adopted to explore in greater depth how speaking up was understood and enacted within a relatively new academic healthcare system. Data collection took place between April and July 2025. All participants provided written informed consent prior to participation. Interviews lasted approximately 45–60 minutes, were audio‐recorded and transcribed via Microsoft Teams. Interviews followed an open‐ended, semi‐structured format in which questions were flexibly adapted as conversations unfolded. In line with a social constructionist orientation, the interviewer adopted a ‘talking back’, stance engaging in a dialogic exchange rather than a purely extractive questioning style (Ruslin et al. 2022). This approach avoided directive or closed questions and instead encouraged participants to elaborate, clarify or complicate their accounts. The intent was to co‐construct meaning through interaction and to explore the cultural, moral and relational nuances embedded in participants' descriptions of speaking up.

Each participant was assigned a pseudonym, accessible only to the principal investigator (S.S.G.), ensuring that no other researcher could link voices to identities. To prevent inadvertent recognition, only S.S.G. accessed the audio files and engaged independently with each transcript, listening, reading and re‐reading the accounts while keeping analytic notes that captured discursive patterns, contradictions and culturally shaped meaning‐making.

### 2.5 Analytical Approach

Critical discourse analysis (CDA) constituted the primary analytic approach, informed by a social constructionist epistemology and a Foucauldian understanding of discourse (Kincheloe and McLaren 2011; Power 2011). Here, power was understood not as something inherently possessed by particular individuals, but as ‘circuitous’—continuously in flux through professional hierarchies, institutional norms, cultural expectations and competing forms of knowledge (Khan and MacEachen 2021). From this perspective, we inferred that meanings do not carry equal legitimacy; rather, individuals draw power from the resources they have access to and can make possible what can be said, known and heard, and which speakers claim and forms of knowledge become socially legitimate or are marginalised or rendered inaudible. This orientation directed our attention to difference, absence and local context rather than to similarity, presence and universal explanations. We were interested not simply in whether HCPs spoke up or remained silent, but in how they constructed the meaning, legitimacy, risks and responsibilities associated with speaking up through language. Analysis therefore attended to the linguistic and rhetorical resources participants drew upon, including pronoun use, modal verbs, metaphors, hedging, legitimacy claims, subject positioning and constructions of power. Particular attention was paid to how participants justified voice or silence, how authority and expertise were negotiated and how professional identities, responsibilities and hierarchies were constructed through talk. Analysis proceeded iteratively through repeated reading and re‐reading of transcripts. Using the sensitising questions outlined in Supplemental Information 1, we examined how participants framed speaking up, justified action or inaction, positioned themselves in relation to others and invoked particular moral, professional, cultural or relational logics (Gee 2025; Sukhera et al. 2022). These sensitised our research team to a critical, Foucauldian‐informed approach while remaining attentive to participants' situated reasoning. Through this process, we identified eight interpretive repertoires; recurrent and culturally recognisable ways of talking about speaking up that appeared across participants' accounts. These repertoires captured the discursive resources through which voice and silence were made intelligible, legitimate or problematic within clinical practice.

Although the interpretive repertoires provided a detailed account of how participants constructed speaking up in specific situations, they remained analytically fragmented (Batchelor et al. 2025). Several repertoires appeared to draw upon similar moral, cultural and professional logics despite differing in their surface linguistic features. Consequently, an interpretive bricolage lens grounded in critical hermeneutics (Kincheloe and McLaren 2011) was employed as a second‐order synthesis strategy. Consistent with bricolage approaches, this involved moving iteratively across repertoires, connecting multiple interpretive perspectives and examining how apparently distinct ways of talking about speaking up converged around broader systems of meaning (Batchelor et al. 2025; Kincheloe 2005; Kincheloe and McLaren 2011). For example, Repertoire I—patriotic stewardship used collective language such as ‘we’, ‘our’, ‘country’ and ‘cultural values’. While Repertoire V—respect, harmony and relational navigation used hedging, indirectness, attention to face and trusted intermediaries. Although their surface language differed, both drew on a deeper moral‐cultural logic of loyalty and protection. Here, speaking up became legitimate when it safeguarded people, relationships, institutions or the wider system rather than appearing confrontational. Importantly, bricolage did not generate additional codes or themes. Rather, it enabled the integration and synthesis of discourse analytic findings across repertoires, revealing the broader cultural‐moral logics organising participants' accounts. By weaving together recurrent strands of meaning, it revealed the deeper cultural‐moral grammars that connected otherwise fragmented discourse analytic findings and illuminated the broader systems of meaning organising participants' accounts (Batchelor et al. 2025).

**Figure 1:**
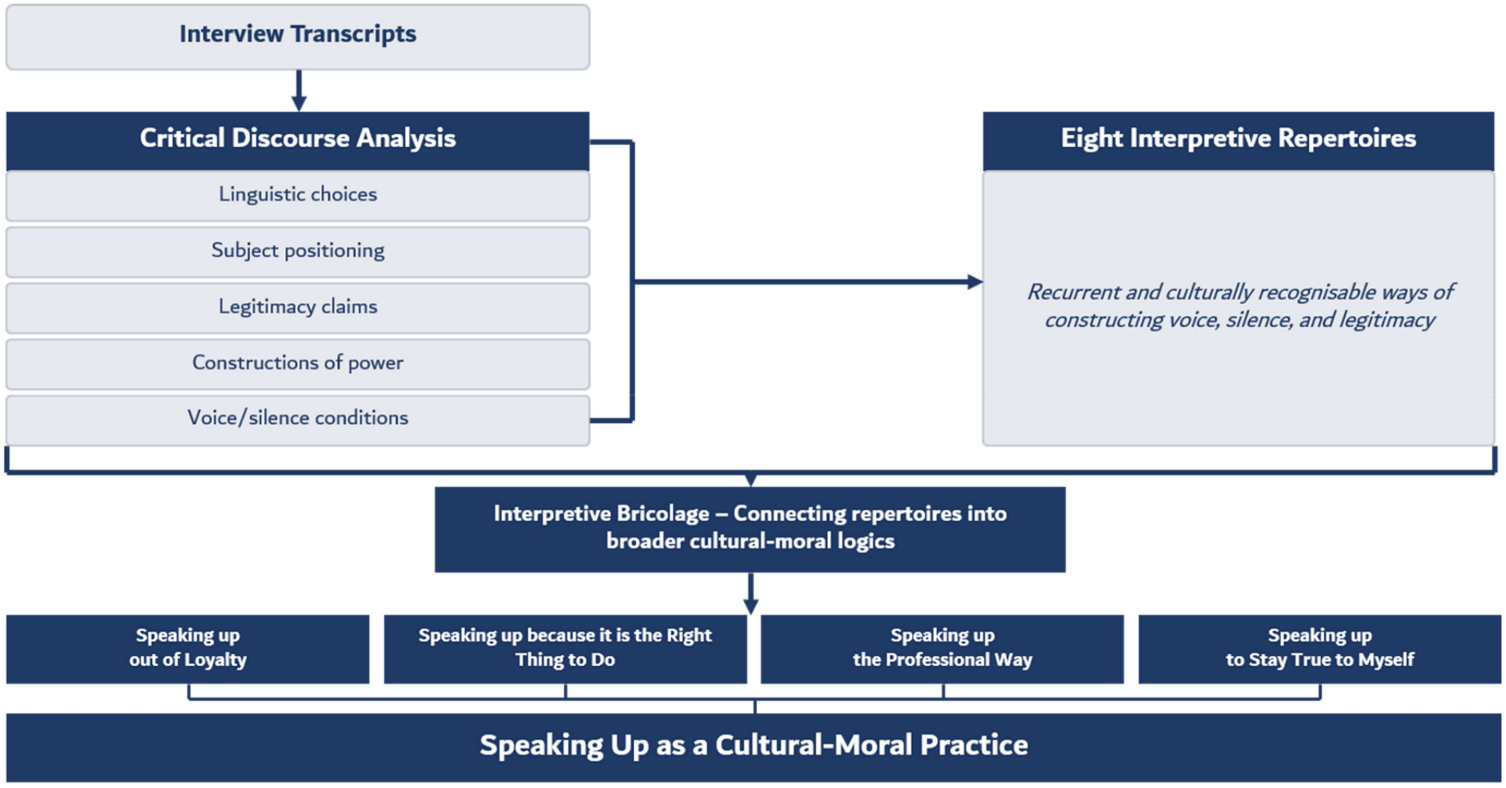
Analytical process: speaking up as a cultural‐moral practice.

Through this interpretive synthesis, the eight repertoires were organised into four higher order definitional discourses. Whereas repertoires captured situated ways of talking about speaking up, the definitional discourses represented broader frameworks of meaning that helped us understand how participants negotiated responsibility, legitimacy, professionalism, loyalty and selfhood. Figure 1 presents a visual overview of the analytical process, illustrating how CDA informed the development of interpretive repertoires, which were subsequently synthesised through interpretive bricolage into four higher order definitional discourses.

### 2.6 Member Checking

Given the sensitive and morally charged nature of speaking up within hierarchical clinical environments, member checking was undertaken with particular intent. Participants' accounts often touched on moments of hesitation, calculation and consequence, making it important that their experiences were not misrepresented or stripped of nuance in the analytic process. Member checking, therefore, involved returning brief interpretive summaries outlining how participants' accounts were being understood within the broader discursive framing of our study. Consistent with our epistemological orientation, this step was approached as a dialogic and ethical practice rather than a procedural test of validity. Decisions regarding its use were guided by careful consideration of our epistemological alignment, participant burden and relational power dynamics (Varpio et al. 2017). All 17 participants were contacted, and no amendments were requested. The analytic framework was therefore not altered; importantly, the absence of revisions was documented and not assumed to indicate agreement with the interpretations.

### 2.7 Reflexivity

See Box 1 for a reflexive account of the research team and our positionalities.

Box 1 **Reflexivity and positionality of the research team.**

**Table:** 

In discourse analysis, meaning is constructed through language but also interpreted through the perspectives of those engaging with the data. Recognising knowledge as co‐constructed, the research team approached analysis as an interpretive process shaped by disciplinary backgrounds, professional roles and sociocultural positioning. The team comprised members with both insider and outsider relationships to the setting.
H.M.K.A. and E.A.H., HCPs native to the region, contributed cultural grounding and contextual interpretation. F.E., locally raised and enculturated, supported the interpretation of implicit norms. I.I., with over three decades of professional experience in the Gulf, provided institutional and historical contextualisation. M.A.H., a senior academic leader with clinical training in dentistry, offered insight into professional hierarchy and interprofessional relations. N.Z., an experienced medical educator, supported the interpretation of discursive framing within health professions education. S.S.G., an Asian female academic and former radiologist who has worked in the Gulf region for over 20 years across clinical and academic roles, brought experiential familiarity with hierarchical communication practices in healthcare. S.S.G. conducted the primary analysis, including repeated engagement with transcripts, development of interpretive repertoires and the subsequent synthesis of findings. Emerging interpretations, analytic memos and developing constructions were discussed iteratively with the wider research team, whose diverse cultural, clinical and educational perspectives were used to interrogate assumptions, explore alternative readings and refine the final analytic framework. These positionings enabled sensitivity to linguistic registers of deference, justification and accountability often embedded subtly within participants' accounts. At the same time, analytic interpretations were continuously examined within the team to ensure that experiential resonance informed attention to the data rather than predetermined conclusions. Analytic notes, interpretations and emerging constructions were discussed iteratively. Through dialogic engagement, team members questioned, refined and challenged interpretations in a non‐hierarchical manner, producing an auditable analytic trail. This process supported an interpretive‐critical marriage, a bricoleur analytic stance, oscillating between interpretive understanding of participants' reasoning and critical examination of how accounts reproduced or resisted professional norms. Considering that most team members were expatriates while two were native to the region, multiple vantage points allowed discursive interpretations to be examined across differing relationships to the cultural context, strengthening credibility and cultural sensitivity of the final analysis. Although bricolage can extend across methodological and theoretical domains, here it operated at the interpretive level only, shaping how accounts were connected to wider institutional expectations rather than serving as a multi‐method approach (Wyatt and Zaidi 2022).

.

### 2.8 Ethical Approval

Ethical approval was granted by Mohammed Bin Rashid University Institutional Review Board (Reference #MBRU IRB‐2025‐5) and by the Dubai Scientific Research Ethics Committee, Dubai Health Authority (DSREC‐02/2025_40). This observational study was conducted in accordance with the ethical standards outlined in the Declaration of Helsinki (General Assembly of the World Medical Association, 2014). Written informed consent was obtained from all participants.

## 3 Results Part 1: Definitional Discourses of Speaking Up

Across participants' accounts, speaking up did not appear as a single or self‐evident behaviour. Instead, it emerged as a morally and relationally situated practice whose meaning shifted depending on who was speaking, to whom and under what conditions. Speaking up was rarely described in purely practical terms; rather, participants spoke about it through values such as loyalty, conscience, professionalism and self‐integrity. What counted as ‘good’, ‘appropriate’ or even ‘possible’ speaking up was therefore neither universal nor fixed, but negotiated in relation to expectations, relationships and perceived consequences.

**Figure 2:**
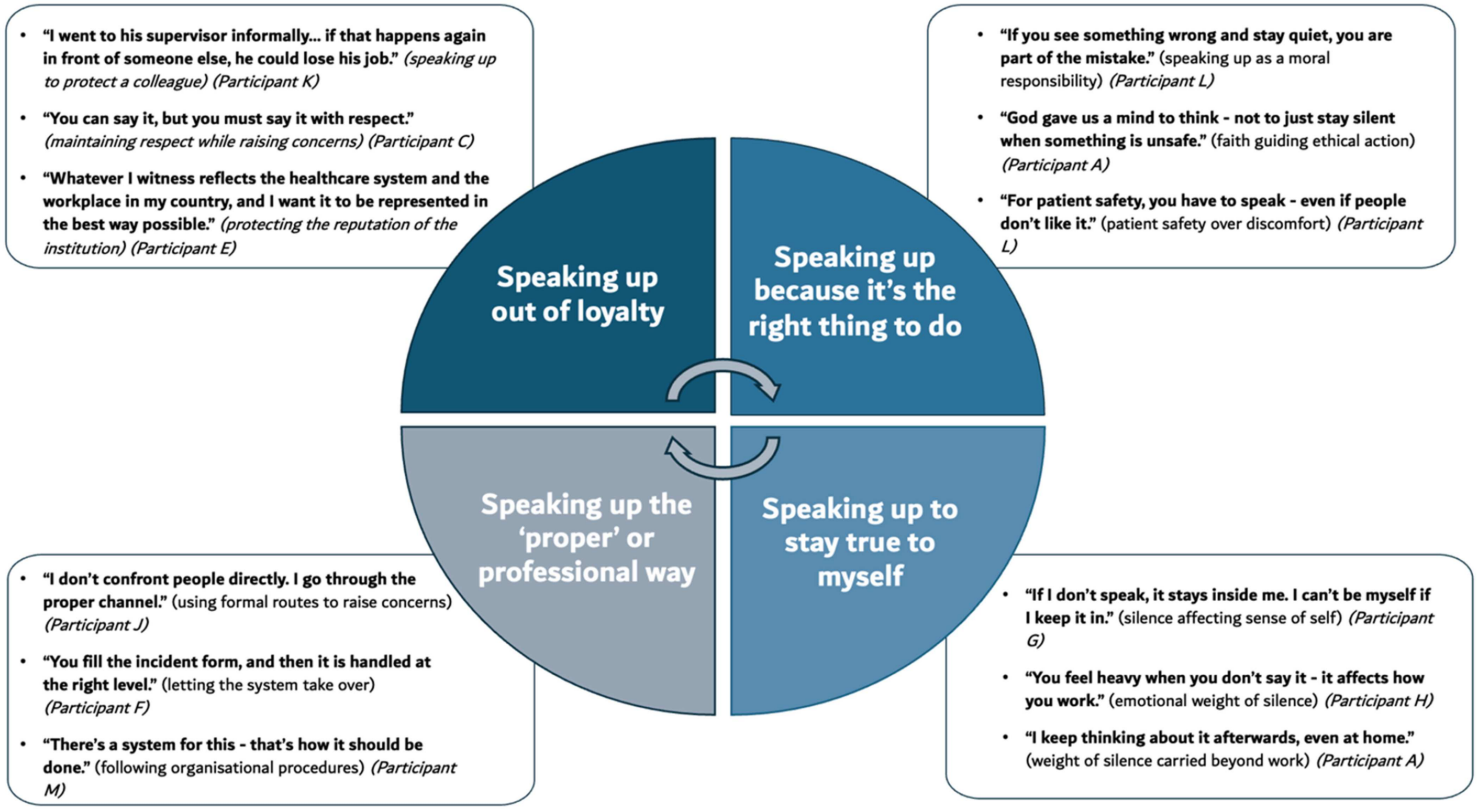
Four overlapping definitional discourses of speaking up as identified in our analysis.

Our analysis identified four overlapping definitional discourses; recurring ways participants made sense of voice and silence. These were not stable categories. Individuals moved between them, sometimes within the same account, drawing on different moral logics as situations, audiences and power relations shifted. We present these discourses using language closely aligned with participants' own terms: (1) speaking up out of loyalty, (2) speaking up because it is the right thing to do, (3) speaking up the ‘proper’ or professional way and (4) speaking up to stay true to myself (see Figure 2).

These discourses are analytic constructs rather than participant types. Taken together, they reflect different ways responsibility was understood: at times grounded in protecting relationships and maintaining order, and at other times in personal integrity and inner consistency. They are neither hierarchical nor developmental, but co‐existing moral positions that participants navigated as they decided whether, how and when to speak. Below, we describe each discourse in turn and what it made possible in practice (see Table 2).

**Table 2:** Description of four definitional discourses of speaking up.

**Discourse I—Speaking up out of loyalty**
Speaking up was framed as an act of care and allegiance to colleagues, the institution or the wider system (Farrell et al. 2021). Within this discourse, voice was not positioned as confrontation but as protection. Moral legitimacy rested on preserving dignity, hierarchy and relationships, often through indirect, private or mediated forms of intervention. Speaking up was judged not only by what was said, but by how and through whom it was expressed. Within this framing, silence was not automatically interpreted as avoidance or fear, unlike Morrison's conceptual paper (Morrison 2014) and Farrell's simplified linear understanding of voice and silence (Farrell et al. 2021). It could signal relational wisdom, restraint or an ethic of care aimed at preventing harm to others' reputation, standing or livelihood (Ng et al. 2017). Speaking up out of loyalty, therefore, reframed critique as stewardship: A way of safeguarding people and systems rather than challenging them. In doing so, this discourse made voice possible without jeopardising social cohesion, yet limiting the forms of critique that could be considered legitimate in our context of study.
**Discourse II—Speaking up because it is the right thing to do**
Speaking up was also constructed as a moral obligation grounded in conscience, faith or ethical conviction (Fagan et al. 2021). Here, legitimacy flowed less from relationships and more from an internal sense of right and wrong. Silence risked being interpreted as moral complicity, particularly when patient safety was perceived to be at stake. This discourse positioned the speaker as an ethical agent whose authority derived from principle rather than rank. At the same time, it was not static. Participants often reflected on how experience had reshaped their enactment of moral duty over time. While the ethical imperative to speak remained, it was increasingly tempered by judgement, timing and awareness of consequences. Speaking up because it was ‘the right thing to do’ thus encompassed both moral clarity and, for some, a growing sensitivity to context. This discourse framed speaking up that an HCP is ought to do, rather something optional, making silence harder to justify in face of patient safety at risk.
**Discourse III—Speaking up the ‘proper’ or professional way**
A third definitional discourse framed speaking up as an expression of professionalism, grounded in procedural correctness and communicative competence rather than personal conviction (Jeong and Kim 2023; Lee et al. 2022; Lee et al. 2023). Within this framing, voice was enacted through systems: Reporting pathways, documentation, escalation processes and adherence to institutional norms. Legitimacy came from knowing how the organisation works and how concerns should be raised without disrupting order. Here, speaking up was displaced from the individual onto the institution. Silence was not necessarily fear‐driven; it could reflect waiting for the appropriate mechanism or moment. Speaking up the ‘proper’ way redefined professionalism as system fluency and emotional regulation, positioning the speaker as competent, measured and aligned with organisational expectations. In this way, legitimacy became tied not only to the content of the concerns but also to the mastery and execution of institutional norms, allowing particular forms of voice while marginalising others.
**Discourse IV—Speaking up to stay true to myself**
Finally, speaking up was framed as essential to emotional well‐being and personal coherence. Within this discourse, silence was described as psychologically costly, and voice as a means of preserving inner alignment. Speaking up was not primarily about systems or duties to others, but about maintaining one's sense of self. This framing foregrounded authenticity as a moral good, with emotional language used to describe the burden of unspoken concerns. Voice became a form of self‐care; silence, a source of emotional dissonance. At the same time, this discourse sat in tension with others, particularly in contexts where expressing oneself carried professional or relational risk (Parker et al. 2019). Hence, staying true to oneself became a legitimacy in its own right, both compelling and constraining, an expression of personal perseverance.

### 3.1 Contextual and Cultural Variation

Together, these four definitional discourses illustrate how speaking up carries multiple, sometimes competing, meanings within a multicultural healthcare environment. Participants navigated shifting expectations about loyalty, conscience, professionalism and selfhood, often moving fluidly between them as circumstances changed. These discourses provided the moral landscapes within which participants later drew on more specific interpretive repertoires to justify, delay, redirect or withhold voice shaping what form of voice was perceived legitimate, appropriate or possible in particular situations. In the following section, we turn to these interpretive repertoires to examine how participants actively reasoned, negotiated risk and positioned themselves within these broader definitional frames in their everyday talk about speaking up.

## 4 Results Part 2: Interpretive Repertoires of Speaking Up

An analysis of the interview discourse revealed eight common interpretive repertoires, recurrent ways in which participants constructed and made sense of their speaking‐up intentions. As in other discourse‐analytic work, these repertoires were fluid and overlapping rather than fixed categories. We have provided the detailed discourse‐analytical characteristics of each repertoire—associated linguistic features, forms of legitimisations, power relations and subject positions in Supplemental Information 2.

From a discourse analytic perspective, these repertoires crystallised when we noticed clusters of specific linguistic choices like pronouns, modal verbs, metaphors, time markers and affective descriptors doing similar interactional work across accounts. Each repertoire told us not only ‘what’ participants said about speaking up but also ‘who they could be’ when they spoke, ‘where the problem was located’ and ‘what forms of knowledge’ were treated as legitimate.

The following sections describe each repertoire in turn, highlighting the discursive work it does and indicating how it maps onto the four definitional discourses outlined earlier.

### 4.1 Repertoire I—Speaking Up as Patriotic Stewardship

In our analysis, we identified a repertoire in which Emirati participants framed speaking up as an act of national responsibility. When they spoke about raising concerns, participants frequently drew on inclusive pronouns such as ‘we’ and ‘our’ linking speaking up to service, protection and how the healthcare system represents the country. Concerns were framed as ‘things that need fixing’ rather than as failures of particular individuals. For these participants, speaking up was not described as confrontation or challenge. Instead, it was framed as a way of caring for the nation and its institutions. One that aimed to ensure that healthcare practices reflected well on the UAE and aligned with shared cultural values.

Because whatever I will be witnessing is something that's going to reflect the healthcare system or the workplace in my country, and it's something that I want to be represented in the best way possible and to be established in the most professional way possible. So I think I am more likely to speak up being an Emirati.

(Participant E)

Here, speaking up is linked to national belonging. The motivation to speak does not come from personal disagreement alone, but from a sense that what happens in the workplace reflects on the country as a whole (Farrell et al. 2021). Cultural values were also used to explain why speaking up felt necessary.

…. So if it doesn't match our cultural values, then I would be very likely to speak up.

(Participant E)

This repertoire also shaped how participants positioned themselves. Emirati nationals spoke as insiders; people who felt a responsibility to look after what is ‘ours’. In contrast, expatriate participants often described themselves as guests within the system and were more cautious about claiming this kind of authority. Importantly, Emirati participants did not express this position in terms of superiority. Instead, they spoke through the language of care, responsibility and stewardship. Repeated references to national representations, cultural values and collective responsibility positioned participants as custodians of the healthcare system whose legitimacy to speak up derived from loyalty and stewardship making critique legitimate and allowing concerns to be raised without positioning oneself as oppositional or confrontational. Power was therefore negotiated gently rather than directly challenged, with concerns framed as opportunities for improvement than failure of particular individuals.

### 4.2 Repertoire II—Speaking Up as a Moral Duty

We developed this repertoire through close attention to moments where participants framed speaking up in moral rather than relational or procedural terms. In these accounts, legitimacy did not come from loyalty to the institution or fluency with systems, but from an internal sense of righteousness. Unlike the patriotic stewardship repertoire, where voice was anchored in belonging (‘our system’, ‘our hospital’), this repertoire was anchored in conscience. The question was not who am I to speak? but how can I stay silent when I know this is wrong? This was particularly evident when participants spoke about fairness, patient vulnerability and moral discomfort, even when they did not ultimately act on that discomfort.

because that's the whole point. But I think it's easier said than done because you have to unlearn a lot of, like natural instincts. But ultimately, ideally yes. I feel like you have to do what's right for the patient despite it being uncomfortable for yourself.

(Participant A)

Here, speaking up is framed as a moral obligation that overrides personal unease. Silence is acknowledged as understandable, but ethically insufficient. For some participants, this moral stance was explicitly linked to religious accountability, where speaking up was framed as a duty before God rather than before supervisors.

This is a very heavy question because I feel that I'm presenting now Islam, but no, we are influenced to speak up.

(Participant L)

Language in this repertoire was characterised by moral absolutes such as ‘right’ and ‘wrong’, obligation‐oriented expressions such as ‘must’ and ‘should’, and, for some participants, explicit references to faith and spiritual accountability. By privileging moral and spiritual knowledge as the highest source of legitimacy, this repertoire positioned participants as ethical actors whose authority to speak up is derived from conscience rather than hierarchy. Speaking up became morally warranted even in the face of personal discomfort and organisational risk and making silence increasingly difficult to justify. Problematisation was sharp and personal: it was not only unsafe or unfair practice that was called into question, but also the possibility of knowing better and remaining silent.

### 4.3 Repertoire III—Speaking Up as Reflective Learning

Moving forward, we identified a third way of talking about speaking up that framed it as something people grow into, rather than something they either possess or lack. Participants frequently used temporal phrases such as ‘before’, ‘earlier’, ‘now’ and ‘these days’ alongside reflective verbs such as ‘learned’, ‘realised’ and ‘understood’. Through this language, speaking up was constructed as a developmental practice, where experience generated not only confidence to speak but also discernment about when, how and whether concerns should be voiced (Beament and Mercer 2016). Speaking up, in these accounts, was described as a practice shaped by qualifications, experience (Nembhard et al. 2015), reflection and learning from past missteps.

I'd say definitely as we age and regain more educational experience, we gain more work experience. I think there has been a shift towards. Speaking up more or holding myself more accountable and the others around me.

(Participant F)

This repertoire was especially common among mid to senior‐level HCPs. Rather than presenting speaking up as an immediate moral obligation or a straightforward act of loyalty, participants described it as something that requires judgement. They spoke about learning when to pause, how to phrase concerns and which moments were worth acting on. Impulsive reactions were often positioned as something of the past, replaced by more thoughtful and calibrated responses.

Like sometimes it's just a verbal discussion with the other person.

(Participant C)

Here, speaking up was constructed as a form of professional apprenticeship, something refined through observation, trial and error, and experience within the system (Landgren et al. 2016).

Unlike the moral duty repertoire, where speaking up was framed as something one must do, this repertoire emphasised timing and delivery. Speaking up was not rejected, but it was no longer seen as automatically virtuous. What mattered was how and when concerns were raised. Silence, in this framing, was not necessarily avoidance; it could signal emotional regulation, restraint or strategic judgement.

You learn to choose your battles. Otherwise you will only create resistance.

(Participant A)

Participants described learning to ‘choose their battles’, recognising that not every issue requires immediate voice and that poorly timed or poorly delivered speaking up could create more harm than good. This repertoire reflected a synthesis of moral concern, organisational awareness and relational sensitivity.

Within this repertoire, the reflective practitioner emerged as someone for whom speaking up required judgement and discernment, where waiting, pausing and strategic silence formed part of responsible action. In this framing, reactive, impulsive and emotionally driven voice were positioned as less effective and potentially detrimental to the individual and system.

### 4.4 Repertoire IV—Speaking Up as Communicative Competence

Fourth, this repertoire framed speaking up as less of a question of whether to speak and more of a question of how to do it (Jeong and Kim 2023; Landgren et al. 2016; D. L. B. Schwappach and Gehring 2015). We began to recognise this repertoire when participants repeatedly focused on tone, wording, timing and emotional control. Rather than emphasising the content of what was said, participants foregrounded the manner of speaking itself. Across both junior and senior participants, speaking up was described as a learned communicative skill. Cultural background also played an important role. Several participants from Arab or Arab‐mixed backgrounds described learning how to navigate hierarchy through politeness and indirectness. In these accounts, moral intention alone was not enough. What mattered was the ability to regulate emotion, choose words carefully and deliver concerns in a way that would be heard without escalating conflict.

You have to say it the right way never in anger. Even even, difficult feedback can be given nicely you know, if you choose your words carefully.

(Participant N)

Language in this repertoire frequently revolved around verbs such as say, talk, approach and explain, alongside affective qualifiers like calmly, gently and respectfully. Participants also spoke about waiting, pausing and choosing the ‘right time’. Through this talk, communication itself became a moral practice. Speaking up was presented as evidence of intelligence, professionalism and emotional maturity. For several participants, especially mid‐career HCPs, emotional regulation was positioned as a marker of growth.

A junior resident, reflecting on an incident he found ethically troubling, explained why he did not speak up immediately, where delay was not framed as avoidance, but as restraint.

During the moment, So it was, it was like I was in a night shift, so it was after hours for him and I just waited till the next morning to when we were in person. I caught him in person and asked him.

(Participant F)

In these accounts, communicative competence emerged as a source of legitimacy, positioning participants as skilled communicators capable of navigating social expectations through diplomacy and emotional regulation. Speaking up therefore became not simply an act of expression, but a demonstration of communicative competence and relational diplomacy. In this repertoire, overt displays of anger, confrontation or emotional intensity were consistently portrayed as unprofessional, even when concerns were morally justified. Participants suggested that speaking up ‘in the wrong way’ could undermine both the message and the relationship. As a result, silence—or delayed speech—was sometimes reframed as a strategic choice rather than a failure of courage.

### 4.5 Repertoire V—Speaking Up as Respect, Harmony and Relational Navigation

In our analysis, we identified a way of talking about speaking up characterised by hedging, indirectness, references to respect caution and social fit (Ng et al. 2017). These functioned not merely as politeness strategies but as relational practices through which participants navigated hierarchy and relationships.

That that speaking up is is essential, and this is right and things. But again within the boundary where you don't exceed. You know, you know, in public doing it, its wrong.

(Participant B)

Rather than positioning voice as an individual right, participants frequently described speaking up as a relational responsibility informed by experience, seniority and social context. Legitimacy to speak up was therefore often distributed rather than individualised, with trusted intermediaries serving as culturally acceptable pathways for raising concerns. A native participant explicitly linked communicative tact to cultural expectations (Lee et al. 2022).

In our culture, if if you know someone well you know, you do not go straight to formal channels. You're expected to approach them first.

(Participant N)

Speaking up, here, was shaped by attention to face, seniority and the social consequences of public critique. This repertoire was especially common among participants with longer clinical experience and among those working within strongly collectivist cultural frames. Participants spoke about the risk of embarrassing others and the need to protect colleagues, even when addressing unsafe or inappropriate behaviour. The concern is clearly voiced, but it is routed through a senior intermediary rather than addressed directly. Although the term ‘wasta’ was not always explicitly named, its logic was clearly present. Speaking up was frequently described as involving trusted individuals who could carry the message more effectively and with less harm, legitimising the distributed nature of speaking up rather than individualised. In these accounts, ‘wasta’ operates as a face‐saving pathway. Several framed this as a way of fixing problems quietly and preventing harm, rather than avoiding responsibility.

I mean but but I just say you know, because people maybe are maybe more cautious to to in this part of the world, maybe not.…you know, the the conservative,… it's more of the, you know, respect of others,… maybe because of the culture of our our culture, how we have brought up, you know.

(Participant B)

Within this repertoire, the relational navigator balanced patient safety concerns against the potential relational consequences of public criticism. Speaking up was framed as a shared act, distributed across relationships rather than performed by a single individual. Silence was therefore not always interpreted as fear or avoidance. Instead, knowing when not to speak, or how not to speak directly, was frequently constructed as a sign of judgement, maturity and cultural intelligence (Dyne et al. 2003).

### 4.6 Repertoire VI—Speaking Up as Procedural Professionalism

Next, we identified a repertoire in which speaking up was framed less as a moral or relational act and more as a technical, procedural task (Jeong and Kim 2023). This way of talking became evident when participants shifted into the language of systems, policies and reporting mechanisms, often using impersonal or passive constructions that placed the institution, rather than the individual, at the centre of action. The emphasis was not on confronting people, but on following processes.

You are going to talk to the through proper channel, to the person who is in charge. Yeah, exactly. The the one who is more having more power and they are more, they're more familiar with the system and they know how the things can be, I mean, facilitated. Such people can do it very confidently, but people with less, like, like, like less.

(Participant J)

Here, legitimacy did not come from moral conviction or personal courage, but from procedural correctness. Speaking up was constructed as professional precisely because it was depersonalised. By routing concerns into formal systems, participants distanced themselves from interpersonal conflict and moral exposure.

…If it if it's been seen by you to be done by your senior colleague, then you need to. This is not as per the guidelines.

(Participant P)

Such language subtly relocated responsibility from the speaker to the institution itself (Lee et al. 2022, 2023). The system became the actor, while HCPs were positioned as compliant professionals whose legitimacy derived from following procedure rather than exercising personal judgement. Participants who most often drew on this repertoire were mid‐ to senior‐level HCPs with long‐standing familiarity with institutional processes. Junior‐level participants repeatedly emphasised that speaking up was acceptable and safe only when done formally.

Professionalism was closely tied to process fidelity. Ethical action was understood as doing things ‘the official way’ administratively. Within this repertoire, the problem was constructed less as an individual's behaviour and more as deviation from established procedure. Power was not directly challenged; instead, it was deferred to the system. The solution, therefore, was not dialogue or moral persuasion, but correct routing; filing, escalating and documenting.

There is like an official way to act and to put your, your, your speak up and your notes in the system.

(Participant M)

Procedural and technical knowledge were therefore privileged over emotional, relational and moral deliberation making it the most systematised and least personalised form of voice. Here ethical responsibility is enacted through compliance with institutional process rather than through interpersonal engagement.

### 4.7 Repertoire VII—Speaking Up as Self‐Combustion

We identified another way of talking about speaking up that framed it primarily as a matter of personal risk (Martinez et al. 2017). Participants mainly residents drawing on this repertoire spoke cautiously, often using language that signalled uncertainty, vulnerability and calculation (Creese et al. 2021; Morrow et al. 2016). Use of modal expressions such as ‘might’, ‘could’ and ‘maybe’, alongside references to vague and distant actors such as ‘they’ and ‘the system’ were the linguistic choices indicating uncertainty and limited personal agency. In contrast to the confidence of procedural professionalism, this repertoire constructed speaking up as something that could threaten one's reputation, relationships or future security (Beament and Mercer 2016). Here, silence was not framed as moral failure but as a deliberate strategy for survival. Participants justified restraint by anticipating consequences that were perceived as too costly or unpredictable making silence a temporally bounded and purposeful phenomenon (Landgren et al. 2016).

100% fear of consequences. Mainly I would say, because as you're become as we're becoming more and more senior, we're like, you know what we have one more year. It's OK. Don't say anything.

(Participant H)

Speaking up is postponed, not because the concern lacks importance, but because endurance is seen as the safer option. Elsewhere, this participant made this logic even more explicit:

We're about to finish. Maybe we shouldn't say anything. It just suck it up and do it, you know.

(Participant H)

The fear here is not of speaking wrongly, but of being read wrongly. Once spoken, words are imagined as irreversible, carrying consequences beyond the speaker's control.

If I speak up, I don't know who will support me.

(Participant I)

Power is experienced as asymmetrical and potentially punitive. Responsibility is externalised ‘they decide’, ‘the system reacts’ and the speaker is positioned as exposed rather than empowered. Articulating this sense of vulnerability in relation to support structures making this repertoire sits discursively in tension with all four definitional discourses. It constrains loyalty, interrupts moral duty, undermines procedural confidence and even complicates authenticity. Yet it also quietly overlaps with speaking up to stay true to myself, insofar as self‐preservation becomes a way of protecting dignity, livelihood and peace of mind. In doing so, this repertoire highlights a critical absence in the system. The lack of genuine psychological safety felt by the junior participants, even where formal mechanisms for voice are said to exist. Within this repertoire, participants were positioned as vulnerable actors whose agency is constrained by hierarchical distance and anticipated consequences making the unsafe conditions as the main problem.

….I'm too junior to to do anything. Why would they listen? How would this make a difference?

(Participant L)

Silence therefore functioned as a protective and purposeful strategy grounded in experiential knowledge of what might happen when concerns are raised.

### 4.8 Repertoire VIII—Speaking Up as Emotional Authenticity

Lastly, we identified a small but compelling way of talking about speaking up that centred not on systems, hierarchy or professional rules but on the self (Fagan et al. 2021; Scanlan and Still 2019). Linguistically, this repertoire was characterised by emotionally saturated language: first‐person pronouns (I, me), affective descriptors (heavy, uncomfortable) and embodied metaphors (inside, stays with you). These linguistic choices positioned participants as authentic selves whose legitimacy to speak derived from emotional integrity rather than professional status, procedural knowledge or moral authority (Rainer and Schneider 2020). Speaking up was framed as necessary for inner alignment rather than compliance with external expectations. For these participants, speaking up was not primarily about policy, duty or procedure. It was about being able to live with oneself.

….I should speak now or I will never speak up.

(Participant G)

Here, silence is not neutral. It actively disrupts the speaker's sense of self. Speaking up becomes a way of preserving emotional integrity rather than asserting authority or fulfilling obligation, linking silence to emotional strain.

I think I've just grown up in in the workspace. I feel like if you don't speak up, if you don't do what's right for you or for your patients in a on a chronic basis, this will, you know, eat you up. If you don't do it, you'll go.

(Participant H)

In such accounts, emotional experience itself becomes the basis for action. Speaking up is constructed as emotionally necessary, almost cathartic, rather than as a calculated ethical or professional move.

……You will not be actively involved in the rest of the patient's management because you feel like, OK, I'm gonna make you become insecure.

(Participant H)

Notably, this repertoire was articulated exclusively by female participants across different cultural and professional backgrounds. This relatively rare repertoire is analytically significant precisely because of its scarcity.

At the same time, this way of speaking was not universally endorsed. Elsewhere in the data, older participants and supervisors occasionally framed such emotional candour as immaturity or lack of control, revealing generational tensions about what counts as acceptable professional expression. Yet for those who articulated it, emotional authenticity functioned as a quiet counter‐discourse to emotional silencing in healthcare. It reveals how narrowly emotional self‐expression is sanctioned within clinical practice, and how speaking up as an act of emotional coherence remains a fragile and gendered form of legitimacy.

## 5 Discussion

Much like professionalism, the ontological status of ‘speaking up’ in healthcare is far from settled. Yet educational and policy discourse often treats it as a self‐evident, measurable behaviour (Dyne et al. 2003; D. Schwappach and Richard 2018; van Dongen et al. 2024). This study instead asked what speaking up actually means in contemporary multicultural clinical practice. Our findings indicate that speaking up is not a single behaviour or stable professional skill, but a morally and relationally situated practice shaped by hierarchy, institutional expectations and lived experience. Participants drew on four definitional discourses (see Figure 2) and a set of eight interpretive repertoires through which they justified, delayed, redirected or withheld voice. These accounts did not represent inconsistency; rather, they reflected different ways HCPs reasoned about responsibility, risk and legitimacy in particular situations. Taken together, the findings suggest that speaking up was neither simply a matter of personal courage nor organisational rules. Participants referred to values such as morality and loyalty, but always in relation to anticipated responses from seniors, colleagues and the institution. Speaking up was therefore shaped by both personal judgement and the broader social and institutional order. This helps explain why voice was enacted unevenly despite shared safety goals. HCPs were not only deciding whether to speak, but whether particular forms of voice would be considered appropriate within the relationships and expectations they inhabited.

### 5.1 What Is Speaking Up?

The question of what it means to ‘speak up’ in healthcare resists simple definition (Martinez et al. 2016; Morrison 2014, 2023; Parker et al. 2019; D. L. B. Schwappach and Gehring 2014; D. L. B. Schwappach and Niederhauser 2019). Across the literature, speaking up has been conceptualised as a moral act, a relational practice, a professional performance and a form of self‐preservation (Morrison 2014, 2023; D. L. B. Schwappach and Gehring 2014). These are not competing explanations but partial views of a multi‐layered phenomenon shaped by personal values, enacted within interpersonal encounters and constrained by organisational and institutional contexts (van Dongen et al. 2024). No single lens, whether moral courage, assertiveness, professionalism or communication skill, adequately captures what speaking up is in practice. This conceptual plurality makes speaking up difficult to theorise without reduction. Behavioural frameworks such as the Theory of Planned Behavior (Guraya et al. 2024; Premeaux 2001) position voice as the product of attitudes, norms and control. While heuristically useful, our findings caution against treating speaking up as a one‐dimensional decision process. Participants' accounts, particularly those of residents, often the most vulnerable within the medical hierarchy needing to *have the bandwidth* to face the risks and emotional labour, illustrate this clearly (Nichol et al. 2024). For this group, speaking up was not experienced as intention translated into action but as a potentially self‐destructive act, repeatedly described as ‘self‐combustion’. Concerns were not merely unheard; voicing them risked reputational harm, relational rupture or subtle sanction, a form of ‘shooting the messenger’. These accounts trouble linear models and instead foreground power, dependency and evaluative hierarchy as determinants of cost. Speaking up, therefore, appears to operate less as behaviour and more as situated judgement. Voice is filtered through timing, hierarchy, relational consequence and anticipated response (Beament and Mercer 2016; Creese et al. 2021; Landgren et al. 2016; Martinez et al. 2017), while silence may represent restraint, prosocial intent or contextual wisdom rather than disengagement and fear (Jeong and Kim 2023). Our findings extend this literature by showing how such judgements are discursively produced in situ, through different forms of legitimacy, subject positions and moral reasoning, reinforcing the danger of treating voice and silence as opposite ends of a continuum (Dyne et al. 2003; Edmondson and Besieux 2021; Kepplinger et al. 2024; Morrison 2011, 2014). Consistent with Kane et al. (2023) definition, speaking up remained grounded in expressing concerns to prevent harm or improve care. Yet enactment varied markedly across groups. Emirati HCPs frequently drew on discourses of stewardship and insider responsibility; long‐enculturated Arab HCPs often emphasised respect, harmony and relational navigation; expatriate HCPs frequently depersonalised voice through procedures to manage relational and organisational risk. Over time, constrained voice was experienced as emotionally corrosive, intersecting with burnout rather than merely reflecting it (Fagan et al. 2021; Scanlan and Still 2019).

A distinctive contribution of this study is the articulation of ‘wasta’ as a legitimate pathway of speaking up rather than a deviation from it. Participants described a trusted, kind‐hearted insider through whom concerns could be raised quietly. This mirrors communication practices in other collectivist cultures, such as implicit communication, politeness, insider orientation and face‐saving strategies (Gao 1998), as well as zhongyong‐based ‘harmony voice’ that preserves relational balance while enabling action (Qu et al. 2018). Here, voice operates through relational legitimacy rather than visibility. ‘Wasta’, therefore, does not represent avoidance but a social capital, an ethically intelligible mechanism for action without confrontation. These findings further illustrate why stabilising speaking up as a discrete behaviour may flatten a socially embedded practice into an individual attribute (Farrell et al. 2021; Lee et al. 2023; Nembhard et al. 2015; D. L. B. Schwappach and Gehring 2015). For nursing and healthcare educators, these findings nudge us to conceptualise speaking up as culturally and relationally negotiated practice rather than solely as an individual communication skill (Lee et al. 2022, 2023; Rainer and Schneider 2020).

### 5.2 Speaking Up in Education and Policy—A Move Beyond Individual and Ritual Practice

Speaking up continues to be treated in educational and policy discourse as something individuals either do or fail to do, and which can be improved through training, exhortation or reporting mechanisms (A. R. J. Kim et al. 2024; D. L. B. Schwappach and Niederhauser 2019). Consequently, the literature has become saturated with intervention‐focused approaches targeting communication skills, leadership behaviours, organisational policies and psychological safety (O'Donovan and McAuliffe 2020). Systematic reviews by Jones et al. (2021) and O'Donovan and McAuliffe (2020) demonstrated the limited and ephemeral interventions, noting how efforts to promote voice are routinely usurped by hierarchy (A. R. J. Kim et al. 2024), collectivist cultural norms (‘social distance’ and ‘closeness of relationships’) (Omura et al. 2018) and institutional responses (Levine et al. 2020).

In response, the field has tended to move between two explanatory poles. At the individual level, communicative competence is proposed as the solution: if HCPs understand team development, they learn to speak politely, assertively and with emotional intelligence, voice will improve (Shuffler et al. 2018). At the organisational level, silence is attributed to deficient climates, and psychological safety is positioned as the primary theory of change (O'Donovan and McAuliffe 2020). Our findings suggest that both positions are incomplete. Participants experienced psychological safety as conditional, interactional and easily withdrawn, sometimes undone by a raised eyebrow, a pause or an ambiguous response (O'Leary 2016). Rather than a stable organisational condition (Morrow et al. 2016), psychological safety appeared fragile and may at times be iatrogenic, increasing vigilance and self‐monitoring when institutional assurances were contradicted by lived experience (Vunnam 2026). Micro‐withdrawals of legitimacy shifted risk back onto individuals, rendering silence protective and voice costly (Parker et al. 2019). However, our data reveal that legitimacy is not fixed but continually negotiated through hierarchy, relationships, professional standing and cultural expectations. Similarly, communicative competence mattered but did not resolve the problem. While some participants drew on repertoires of communicative competence and relational diplomacy, residents continued to experience speaking up as a form of self‐combustion regardless of their skill, intention or preparation. These professionals described being structurally embedded within hierarchies of evaluation and dependency where the cost of speaking outweighed any communicative advantage (Nichol et al. 2024). Improving how individuals speak in such contexts may do little to change whether they can speak at all.

Although contemporary literature has rightly moved beyond blaming individuals, it often reconstitutes the organisation as the sole culprit (Kepplinger et al. 2024; Morrow et al. 2016; Umoren et al. 2022). Our findings suggest a broader interpretation. Speaking up is neither merely an individual behaviour nor solely an organisational failure. For nursing and healthcare educators, these findings suggest that educational interventions, therefore, risk acting as blunt instruments when applied to deeply rooted social practices. Even where psychological safety initiatives emphasise ‘mutual respect’, the problem becomes a moving ‘sweet spot’. Too little respect suppresses voice, but too much slides into deference shaped by hierarchy and culture (Brown and McCormack 2016; Gao 1998; Qu et al. 2018). In such conditions, improving communicative technique or declaring psychological safety may alter rhetoric more than practice, leaving the underlying moral negotiations of voice largely untouched (Okuyama et al. 2014). Attempts to address an ontologically chameleon concept, such as speaking up, by recasting it into another abstract construct, psychological safety, may result in a shotgun approach, effectively measuring one indeterminate phenomenon with another (Shuffler et al. 2018).

## 6 Limitations and Strengths

This study has several limitations that should be considered when interpreting the findings. First, although participants represented a range of clinical disciplines, nationalities, seniority levels and cultural orientations, the sample does not capture the full diversity of HCPs working in multicultural systems. Those who volunteered for in‐depth interviews may have been more reflective or more attuned to speaking up than those who declined to participate, and their accounts may therefore privilege certain moral framings over others. Second, this study focused on professionals working within an academic healthcare setting. Participants' familiarity with educational discourse, reflective practice and institutional language may differ from that of HCPs working in non‐academic or purely service‐driven environments, potentially shaping how speaking up was articulated and legitimised. Third, our analysis examined discursive constructions of speaking up rather than observed behaviour. As is consistent with a social constructionist approach, the aim was not to determine whether participants did or did not speak up, but to understand how they made sense of speaking up, silence, risk and legitimacy through language. While this provides insight into moral reasoning and subject positioning, future work could productively explore how these discourses interact with observed practices over time. Finally, this study centred HCPs' perspectives. Patients' and families' understandings of speaking up, professionalism and silence were beyond the scope of this work but remain important areas for future research, particularly given the relational and moral dimensions highlighted in our findings.

A further consideration relates to the analytic stance adopted in this study. While the work was grounded in CDA, interpretation required movement between participants' situated reasoning and the broader normative structures shaping it. Accounts, therefore, operated simultaneously as personal meaning‐making and as effects of discourse (Wyatt and Zaidi 2022). The analysis adopted a bricoleur orientation, iteratively connecting HCPs' explanations with institutional and professional expectations. Although this approach may appear less procedurally bounded than single‐paradigm approaches, it reflects the complexity of the phenomenon and was managed through reflexive team dialogue and an auditable analytic trail. Future research may further formalise analytic strategies for examining how moral reasoning, legitimacy and discursive regulation interact in speaking‐up practices.

## 7 Conclusion

The descriptions of speaking up articulated by HCPs in this study can be broadly organised within the four definitional discourses identified in our analysis. However, the underlying complexity of these accounts challenges the usefulness of ‘speaking up’ as anything more than a high‐level semantic label. Speaking up, if it has any ontological status at all, may be best understood as a situated construct emerging at the conjunction of self, relationships, culture and institutional context, and as a meta‐practice involving the selection of appropriate communicative strategies rather than the execution of a single behaviour. Rather than attempting to promote speaking up as an abstract, universally desirable act of uncertain meaning, educational and organisational initiatives may be better served by focusing on the development of contextual judgement, relational awareness and organisational conditions that sustain voice without converting it into an act of exposure. In this sense, the issue is rarely a deficit of courage. HCPs were morally willing, but the form, timing and cost of voice varied across contexts. Framing voice and silence as opposite poles, or relying solely on psychological safety and communicative competence as solutions, risks overlooking this situated judgement. What is required is not a stronger exhortation to speak, but a deeper understanding of how HCPs discern when, how and through whom speaking up becomes possible.

## Author Contributions

**Shaista Salman Guraya:** conceptualization, project administration, methodology, data curation, investigation, visualization, formal analysis, funding acquisition, writing – original draft, writing – review and editing. **Farah Ennab:** investigation, methodology, visualization, writing – original draft, writing – review and editing. **Hafidh Mohammad Khamis AlRajaby:** methodology, investigation, writing – review and editing. **Ibrahim Inuwa:** methodology, investigation, writing – review and editing. **Entesar Al Hammadi:** investigation, formal analysis, writing – review and editing. **Manal Al Halabi:** investigation, formal analysis, writing – review and editing. **Nabil Zary:** formal analysis, investigation, methodology, writing – review and editing.

## Ethics Statement

Ethical approval was granted by Mohammed Bin Rashid University Institutional Review Board (Reference #MBRU IRB‐2025‐5) and by the Dubai Scientific Research Ethics Committee, Dubai Health Authority (DSREC‐02/2025_40). This observational study was conducted in accordance with the ethical standards outlined in the Declaration of Helsinki. Written informed consent was obtained from all participants.

## Conflicts of Interest

The authors declare no conflicts of interest.

## Artificial Intelligence Use Statement

The generative AI‐based language tool (Grammarly Pro) was used solely for grammar corrections, readability enhancement and linguistic refinement. All intellectual content, analysis, interpretations and conclusions remain entirely those of the authors.

## Supporting information


Supporting File

## Data Availability

The original contributions presented in the study are included in the article material; further inquiries can be directed to the corresponding author.
