# Supplementary material for: ‘If You Don't Speak Up, It Will Eat You Up’: A Critical Discourse Analysis of Voice and Silence in a Multicultural Clinical Healthcare System
Source: Nurs Inq. 2026 Aug 25;33(4):e70159. doi: 10.1111/nin.70159 (PMC13506097; doi:10.1111/nin.70159)
Supplement: Supplementary file 1 [file NIN-33-e70159-s001.docx]

**Supplemental Information 1**

**Analytic sensitisations guiding the discourse analysis**

| **Analytic focus** | **Analytic questions we asked the data** |
| --- | --- |
| Discursive noticing | What linguistic choices (pronouns, modal verbs, metaphors, hedges) are doing interactional work?  How does the speaker frame speaking up; morally, emotionally, culturally?  What is emphasised, softened, avoided, or left unsaid? |
| Interpretive repertoires | What recurrent ways of talking about speaking up appear across participants?  How do speakers draw on culturally recognisable narratives?  When do they shift repertoires, and for what interactional purpose? |
| Subject positioning | What identities become available to the speaker through this discourse?  Who can they be in this narrative?  What identities are constrained or disallowed? |
| Discursive strands | How do different strands of discourse (e.g., respect, fear, professionalism) intersect or contradict each other?  What tensions, inconsistencies, or shifts appear within and between accounts?  What cultural logics underlie these strands? |
| Rhetorical strategies | How do speakers justify or legitimise speaking up or staying silent?  What arguments, moral claims, metaphors, or emotional appeals are used?  How is credibility constructed or defended? |
| Problematization | What is constructed as “the problem” in speaking up?  Who is responsible for addressing or fixing the issue (self, senior, system)?  What gets naturalised as inevitable or unchangeable? |
| Power relations | How is hierarchy invoked, negotiated, or resisted?  How do cultural expectations (collectivist vs. individualist) shape perceived safety or legitimacy of voice?  How do nationality, seniority, or institutional norms appear in the discourse? |
| Knowledge/expertise claims | What forms of knowledge (procedural, moral, emotional, experiential) are privileged or marginalised?  How do speakers claim authority to speak up or to remain silent?  How do institutional norms inform these claims? |
| Discursive synthesis and definitional discourses | What deeper cultural–moral grammars structure these ways of speaking?  How do repertoires cluster into overarching discourses? |

**Supplemental Information 2**

**Eight interpretive repertoires of speaking up with associated language, power, and legitimization attributes as identified in the data.**

| **Repertoire** | **Use of language** | **Power** | **Legitimization** | **Subject positioning** | **Illustrative quotes** |
| --- | --- | --- | --- | --- | --- |
| **I - Speaking up as patriotic stewardship** | Inclusive pronouns (“we,” “our”); verbs of care and improvement (“fix,” “make better”); evaluative language tied to national values and representation | Power is softened and exercised laterally through care rather than confrontation, hierarchy is respected rather than challenged | Legitimised through national belonging, stewardship, and responsibility for how the system reflects the country | Speaker positioned as an insider custodian or guardian of “what is ours,” authorised to speak through loyalty rather than rank | *“I would be offended and that would motivate me to speak up. So for example, this isn't inappropriate whether it's an action, it's a way of addressing. It's a way of behaving in public.*  *So if it doesn't match our cultural values, then I would be very likely to speak up.”* *(Participant E)* |
| **II - Speaking up as a moral duty** | Moral absolutes (“right,” “wrong”); obligation verbs (“must,” “should”); ethical and faith-based references | Power is re-routed away from hierarchy toward conscience or divine accountability | Legitimised through ethical conviction, fairness, and religious or moral responsibility | Speaker positioned as an ethical agent/individual compelled to act regardless of hierarchy | *This is a very heavy question because I feel that I'm presenting now Islam, but no, we are influenced to speak up.*  *Actually, we are influenced to change with our hands if it doesn't. If we can't, then with our tongue. If not, then I mean with our words.*  *If not, then at least refuse it by heart. So it depends on how this is situation is. So we are influenced to speak up and to make changes whenever feasible and doable.” (Participant L)* |
| **III - Speaking up as reflective learning** | Temporal markers (“before,” “now”); reflective verbs (“learned,” “realised”); evaluative comparisons over time | Power is managed through timing, judgment, and restraint rather than immediacy | Legitimised through qualifications, experience, maturity, and contextual wisdom | Speaker positioned as a wise, reflective professional who has learned how and when to speak | *“But I don't speak up for each and everything.*  *Like sometimes it's just a verbal discussion with the other person.”(Participant C)*  *“No, no, I'm more strong now and I'm more confident to speak up now. Maybe 12 years ago I was still new. I was junior. I wouldn't have spoken.” (Participant D)* |
| **IV – Speaking up as communicative competence** | Metacommunicative talk (“how to say it verbally and non verbally”), tone and affect regulation (“calmly,” “politely”); skill-focused language | Power is negotiated through politeness and emotional control | Legitimised through communication skill, emotional regulation, and professionalism | Speaker positioned as a skilled communicator who exercises agency without threatening relationships | *“Or for me to speak up, I have to be loud. I have to be aggressive.*  *I have to be, you know, but NO…. speaking up….You can also be in our lives very diplomatic way without even causing disrespect. Or you know causing like. A scene.” (Participant D)* |
| **V - Speaking up as respect, harmony, and relational navigation** | Hedging (“maybe,” “sometimes”), honorifics, indirect phrasing, references to intermediaries typical of collectivism | Power is navigated relationally and redistributed through trusted and more influential others | Legitimised through cultural knowledge, respect, and face-saving practices | Speaker positioned as a relational navigator who protects dignity while enabling action | *““And I spoke to a supervisor informally.*  *I I told him. Well, this is what he says. This is not allowed this time. It happened in front of me. But if it happens again in front of someone else, he could lose his job.*  *Please speak to him.” (Participant K)*  *“Even if it’s wrong, you cannot embarrass your colleague in front of others.” (Participant K)* |
| **VI - Speaking up as procedural professionalism** | Bureaucratic and procedural vocabulary (“report,” “document,” “escalate”), passive constructions | Power is deferred to the system and institutional processes | Legitimised through policies, protocols, and formal reporting mechanisms | Speaker positioned as a depersonalised compliant institutional actor aligned with system authority | *“That is not the right thing to be done in that case, so that needs to be reported, or at least. If it if it's been seen by you to be done by your senior colleague, then you need to. This is not as per the guidelines.”(Participant P)*  *“I will speak up. But I will speak to the proper supervisor. Not the person who did it.” (Participant J)* |
| **VII - Speaking up as self-combustion** | Modal verbs (“might,” “could”), negations, vague agents (“they,” “the system”) risk language | Power is experienced as asymmetrical and potentially punitive | Legitimised through survival, risk awareness, and past experience | Speaker positioned as a vulnerable actor prioritising safety, reputation, or livelihood | *“Just makes us feel very helpless because at the end of the day, like what?*  *What can we do if the institution itself is not supporting us in this and so like I mentioned, we resort to kind of backup methods where?*  *You know, we kind of just tell each other. I don't think it's ideal at all.”*  *“If I speak up, I don’t know who will support me.” (Participant I)*  *“I always feel that. Like who am I to speak to, speak it up or like?*  *Why would I like? Why? Why did it come from me?*  *I'm too junior to to do anything ..*  *Why would they listen? How would this make a difference?”*  *(Participant L)* |
| **VIII - Speaking up as emotional authenticity** | First-person language (“I,” “me”); affective terms (“heavy,” “upset”), embodied metaphors | Power is experienced internally rather than institutionally | Legitimised through emotional integrity and wellbeing | Speaker positioned as an authentic self seeking alignment between feelings and action | *“No, I I am a bold and a direct person.*  *So I was not scared and I think I I felt at that point is.*  *I should speak now or I will never speak up.” (Participant G)*  *You should speak up for yourself, and I do tell at least my junior. Is that now? Since I've learnt it? So it affects your performance. Let's say you just, you were yelled at at the beginning of the shift. You made a mistake, you know you. You will not be actively involved in the rest of the patient's management because you feel like, OK, I'm gonna make you become insecure.(Participant H)* |
